# Non-Alcoholic Fatty Liver Disease Markers Associated with Fasting Serum Insulin and Urinary Albumin Excretion Independent of Fasting Plasma Glucose

**DOI:** 10.3390/jcm9103161

**Published:** 2020-09-29

**Authors:** Shuichi Katoh, Markku Peltonen, Mikio Zeniya, Yoichi Sakamoto, Kazunori Utsunomiya, Rimei Nishimura, Jaakko Tuomilehto

**Affiliations:** 1 Division of Diabetes, Metabolism & Endocrinology, The Jikei University School of Medicine, 3-25-8 Nishishimbashi, Minato-ku, Tokyo 105-8461, Japan; rimei@jikei.ac.jp; 2 Public Health Promotion Unit, Finnish Institute for Health and Welfare, Mannerheimintie 166, FI-00271 Helsinki, Finland; markku.peltonen@thl.fi (M.P.); jaakko.tuomilehto@thl.fi (J.T.); 3 Gastroenterology, Akasaka Sanno Medical Center, 4-1-26W Akasaka, Minato-ku, Tokyo 107-8402, Japan; zeniya@xk9.so-net.ne.jp; 4 The Jikei University School of Medicine, 3-25-8 Nishishimbashi, Minato-ku, Tokyo 105-8461, Japan; ysaka@jikei.ac.jp; 5 Department of Health-Care Center, The Jikei University School of Medicine, 3-25-8 Nishishimbashi, Minato-ku, Tokyo 105-8461, Japan; kazu-utsunomiya@jikei.ac.jp; 6 Diabetes Research Group, King Abdulaziz University, Jeddah 21589, Saudi Arabia

**Keywords:** non-alcoholic fatty liver disease, fasting serum insulin, urinary albumin excretion, serum alanine aminotransferase, diabetes, impaired fasting glucose

## Abstract

Objective: We examined the association between non-alcoholic fatty liver disease (NAFLD) markers and fasting serum immunoreactive insulin (FIRI) and urinary albumin excretion (UAE). Subjects and methods: This study comprised Periods I and II from January 2007 to May 2009, and from June 2009 to December 2011, respectively. After excluding people with ethanol intake ≥210 g/week in men and ≥140 g/week in women, 961 people (613 men, 348 women; mean age: 44 years) were included. We evaluated the fatty liver using ultrasonography score (FLUS) and measured liver enzymes. Results: The mean observation period was 25 ± 9 months. We stratified people into two groups by fasting plasma glucose (FPG) in Period I. The cutoff point between the lower FPG and higher FPG was 100 mg/dL. In regression analysis, serum alanine aminotransferase (ALT) (*p* < 0.001), FLUS (*p* < 0.001) and γ-glutamyl transpeptidase (GGTP) (*p* = 0.022) in Period I were independently associated with FIRI in Period II, whereas in all participants FPG was not. ALT (*p* < 0.001) and GGTP (*p* = 0.001) were also independently associated with UAE in people with FPG < 100 mg/dL in Period II. Conclusions: Some NAFLD markers were associated with FIRI and UAE independently of fasting plasma glucose.

## 1. Introduction

Several markers have been reported to be risk factors associated with cardiovascular disease and mortality. The Diabetes Epidemiology: Collaborative analysis Of Diagnostic criteria in Europe (DECODE) study group reported that a high fasting serum insulin level was significantly and independently associated with cardiovascular mortality in non-diabetic men and women [1]. Increased urinary albumin excretion (UAE) has been shown to be independently associated with all-cause mortality in the general population, and the relationship was significant even at levels of albuminuria considered to be within the normal range [2]. Despite these interesting findings, it is not always possible to evaluate fasting serum insulin and UAE at routine clinical examinations, and therefore we should look for useful predictors for those parameters. Thus, further studies are needed to investigate associations between those parameters and non-alcoholic fatty liver disease (NAFLD) markers to clarify whether NAFLD affects fasting insulin and UAE independent of glycemia, hypertension, and other confounders. Moreover, NAFLD markers may be usefully predictive of fasting insulin and UAE. We aimed at elucidating such associations in a retrospective cohort study of Japanese people. 

## 2. Materials and Methods

### 2.1. Study Population 

This retrospective cohort analysis included workers and retirees aged 24–73 years who were undergoing an annual medical check-up as part of the health care system at the Jikei University Harumi Triton Clinic Health Care Center, Tokyo, Japan, between January 2007 and December 2011. The comprehensive health check-up was carried out, and the items of examination were determined in accordance with a contract with each person and his/her employer. Periods I and II of the study were defined as January 2007 to May 2009 and June 2009 to December 2011, respectively. A total of 1341 people (919 men, 422 women; mean age: 44.1 ± 8.6 years) underwent an annual medical check-up in both Periods I and II. Exclusion criteria according to the position statement of the European Association for the Study of the Liver (EASL) 2009 special conference [3], were (i) ethanol intake ≥210 g/week in men and ≥140 g/week in women in Period I (306 people were excluded) and/or Period II (233 people were excluded); (ii) hepatitis B surface antigen positivity (*n* = 11) and (iii) hepatitis C antibody positivity (*n* = 1). Some of these were duplicates (Supplemental Figure S1).

Consequently, a total of 961 people (613 men, 348 women; mean age: 43.5 ± 8.5 years) were included in this retrospective analysis. Informed consent to use their health check-up data, and to additionally evaluate fasting insulin and UAE as outcomes in Period II, for this study was obtained from the above 961 people (there were 2 missing values in UAE), as well as cystatin C (*n* = 190) and high-sensitivity C-reactive protein (hs-CRP) (*n* = 175). The numbers for evaluation of standard CRP in Periods I and II were determined by the above contracts. 

Diabetes was diagnosed by (i) a current prescription of glucose-lowering drugs; (ii) fasting plasma glucose (FPG) ≥ 126mg/dL (6.8 mmol/L); (iii) glycated hemoglobin (HbA1c) ≥ 6.5% (47.5 mmol/mol); and/or a self-reported past history of diabetes in those not yet treated with drugs. 

Fatty liver using ultrasonography score (FLUS) was assigned as described in previous studies [4,5]. Briefly, study-specific FLUS was assigned as follows: 2 points, moderate or severe fatty liver (deep attenuation, vascular blurring, a fatty bandless sign and/or a brighter liver compared with the spleen [6,7,8]; 1 point, mild fatty liver (bright liver, focal fatty sparing [6] and/or high hepato-renal echo contrast [7,9]; and 0 points, normal liver. Medical sonographers were registered with the Japan Society of Ultrasonics in Medicine and had been trained at the Jikei University Hospital. They were unaware of the aims of the study and blinded to laboratory data during the ultrasonography examination. The number of people who underwent abdominal ultrasonography was determined by the above-mentioned contracts.

We evaluated the study-specific Japanese diabetes risk score (JPDRISC) [5] by modifying the original Finnish diabetes risk score (FINDRISC) [10] and applying the International Diabetes Federation for the Asia-specific waist circumference criteria for the metabolic syndrome [11]. The JPDRISC comprised information on age, body mass index (BMI), waist circumference, physical activity, dietary habits (vegetables, fruits, or berries), antihypertensive medication, past history of high blood glucose, and family history of diabetes in a parent, brother, sister, or child. (Supplemental Table S1). 

### 2.2. Data Collection 

Information on medical history and lifestyle, including ethanol consumption, was documented by a self-reported questionnaire and an interview by a professional nurse. Height and weight were measured with the person wearing lightweight indoor clothes to calculate BMI as weight/height^2^ (kg/m^2^). Standing waist circumference while the upper trunk was undressed was measured to the nearest centimeter using a non-elastic soft tape measure at the umbilical level, when the umbilicus was between the lowest rib and the level of the iliac crest, or midway between the lowest rib and the level of the iliac crest. Blood pressure was measured after 5 min rest while the participant was sitting before blood sampling. Venous blood specimens (1.8 mL plasma and 12 mL serum) were drawn between 9:00 a.m. and 10:30 a.m. after an overnight fast. For measurements of FPG and HbA1c, blood was collected using blood collection tubes containing sodium fluoride. Plasma and serum were separated within thirty minutes and stored at 10–15 °C prior to analysis that was done within four hours. 

### 2.3. Laboratory Analyses 

FPG levels and HbA1c (following the US National Glycohemoglobin Standardization Program values [12]) were determined by the glucose oxidase immobilized oxygen electrode method (GA08III; A&T Corporation, Kanagawa, Japan) and high-performance liquid chromatography (HLC-723G9; Tosoh Corporation, Tokyo, Japan), respectively. Enzymatic methods were used to analyze serum high-density-lipoprotein (HDL) (MetaboLead HDL-C; Kowa Medex, Tokyo, Japan), aspartate transaminase (AST), alanine transaminase (ALT) (SLT-J2; Wako Pure Chemical Industries, Osaka, Japan), total cholesterol (TC) (Determiner TC II; Kyowa Medix, Tokyo, Japan), and triglycerides (TG) (Cholestest TG; Sekisui Medical, Tokyo, Japan). Serum cholinesterase (ChE) and γ-glutamyl transpeptidase (GGTP) levels were determined using a butyrylthiocholine iodide method and γ-glutamyl-3-carboxy-4-nitroanilide method (both Wako Pure Chemical Industries, Ltd., Osaka, Japan), respectively. Serum hepatitis B virus surface antigen and hepatitis C virus antibody (3rd generation) were quantified by chemiluminescent enzyme immunoassay (CLEIA) (Architect i2000SR; Abbott Japan, Tokyo, Japan). Serum CRP and hs-CRP were quantified by latex agglutination immunoassay (Iatro CRP-EX, Mitsubishi Chemical Medicine Corporation, Tokyo, Japan) and nephelometry (SRL, Inc., Tokyo, Japan), respectively. Serum fasting immunoreactive insulin (FIRI), UAE, and serum cystatin C levels were determined using CLEIA methods, immuno-nephelometry, and colloidal gold agglutination methods (all, SRL, Inc.), respectively. 

### 2.4. Statistical Analysis 

Clinical data of the participants are presented as the mean ± standard deviation (SD) for continuous variables. The association between risk factors (FIRI, UAE, hs-CRP, and CRP) and other variables including fatty liver and glycemic markers were evaluated by linear regression methods. Histograms of the variables are shown in Supplemental Figures S2–S5. Supplemental Table S2 shows skewness and kurtosis of variables of the variables. We evaluated correlation coefficients between FIRI in Period II and variables in Period I (Supplemental Table S3), between UAE in Period II and variables in Period I (Supplemental Table S4), and between hs-CRP in Period II and variables in Period I (Supplemental Table S5). We calculated standardized regression coefficient, beta (β). Each regression coefficient was adjusted according to a ratio of ordinary sample standard deviations, to indicate the relative importance of variables. We used PASW Statistics 18.0.0 software (IBM Japan, Tokyo, Japan, https://www.ibm.com/ibm/jp/en/) for statistical analyses and *p* values less than 0.05 were considered statistically significant. 

### 2.5. Ethics Statement 

The study was approved by the Institutional Review Board of the Jikei University School of Medicine (20-130 5420) and carried out in accordance with the Declaration of Helsinki. All people who provided written informed consent were recruited. 

## 3. Results

### 3.1. Clinical Profiles

This study comprised Periods I and II, from January 2007 to May 2009, and from June 2009 to December 2011, respectively. The clinical profiles of 961 people are shown in Table 1. The overall mean observation period was 25 ± 9 months; 47 and 62 people had diabetes in Period I and Period II, respectively. We stratified people into two groups based on FPG in Period I, and the cutoff point between the lower FPG (<100 mg/dL) and higher FPG (≥100 mg/dL) was that of impaired fasting glucose (IFG) [13]. Age, BMI, HbA1c, waist circumference, systolic and diastolic blood pressure, FLUS, total JPDRISC value, and levels of serum TG, TC, AST, ALT, GGTP, and ChE during Period I were significantly higher in people with FPG ≥ 100 mg/dL than those with FPG < 100 mg/dL, and serum HDL cholesterol was lower. In addition, serum FIRI, UAE, and cystatin C in people with FPG ≥ 100 mg/dL in Period II were significantly higher than in people with FPG < 100 mg/dL (Table 1). 

### 3.2. The Association between Parameters Measured in Period I and FIRI in Period II

We carried out regression analysis, with FIRI in Period II as the dependent variable. ALT (*p* < 0.001), FLUS (*p* < 0.001), and GGTP (*p* = 0.022) as well as JPDRISC (*p* = 0.006) and TG (*p* < 0.001) in Period I were significantly associated with FIRI in all participants. ALT (*p* < 0.001), FLUS (*p* < 0.001), and GGTP (*p* = 0.001) in Period I were significantly and independently associated with FIRI in Period II in people with FPG < 100 mg/dL as was FPG (*p* = 0.010). In people with FPG ≥ 100 mg/dL, FPG, FLUS, or GGTP were not associated with FIRI, whereas ALT (*p* = 0.016), JPDRISC (*p* = 0.001), and TG (*p* = 0.001) were. ALT in Period I was independently associated with FIRI in Period II, in both people with FPG < 100 mg/dL, and those with FPG ≥ 100 mg/dL, whereas FPG was not in all participants (Table 2). Table 3 shows the association between parameters measured in Period II and FIRI in Period II (i.e., cross sectional analysis). 

### 3.3. The Association between Parameters Measured in Period I and UAE in Period II

Table 4 shows results of regression analysis with UAE as the dependent variable in Period II. GGTP (*p* = 0.005) in Period I was significantly and independently associated with UAE in Period II in all people, as was FPG (*p* < 0.001). ALT (*p* < 0.001) in Period I was significantly and independently associated with UAE in people with FPG < 100 mg/dL. In Period I in people with FPG ≥ 100 mg/dL, ALT or GGTP were not associated with UAE, while FPG (*p* < 0.001) and TC (*p* = 0.028) were. Table 5 shows the cross-sectional association between parameters measured in Period II and UAE in Period II. 

### 3.4. The Association between Parameters Measured in Period I and hs-CRP in Period II

In subgroup analyses, FLUS (*p* = 0.008) and TG (*p* < 0.001) in Period I were significantly and independently associated with hs-CRP in Period II in 120 people (94 and 26 people with FPG < 100 mg/dL and FPG ≥ 100, respectively) (Supplemental Table S6). The cross-sectional association between parameters measured in Period II and hs-CRP in Period II is shown in Supplemental Table S7. 

## 4. Discussion

We previously reported that FLUS correlated with increasing HbA1c in 5384 people without diabetes and those with drug-naïve type 2 diabetes in a cross-sectional analysis [4]. We also reported that FLUS and JPDRISC were independently associated with incident diabetes [5]. These results suggested a significant association between NAFLD and the development of type 2 diabetes. A recent study showed that ALT was directly associated with cardiovascular risk factors independent of obesity, even when ALT was within normal range [14]. We therefore speculated that some fatty liver markers might be associated with emerging cardiovascular risk factors such as FIRI and UAE, which in some people are increased before and just after the development of diabetes. Although we demonstrated a significant and independent relationship between fatty liver markers and these cardiovascular risk factors (FIRI and UAE), we could not clarify the mechanism and cause/effect relationships by our present observational study. Based on previous studies, we can suggest a possible mechanism as follows. Decreased insulin clearance in NAFLD might result in an elevation of insulin level in blood circulation [15]. Our results for FIRI were consistent with the hypothesis that ALT within or slightly above the upper limit of normal was associated with fatty metamorphosis of hepatocytes followed by insulin resistance, a previously indicated pathophysiologic process [15,16,17,18,19]. 

Although GGTP is one of the markers of fatty liver, our regression analysis showed a negative beta coefficient between GGTP and FIRI. Since we had excluded people with ethanol intake ≥210 g/week in men and ≥140 g/week in women, this resulted in many people with high GGTP being excluded, and might have paradoxically resulted in this negative beta coefficient when analyzed with ALT and ChE. 

The mean values of UAE were 11.5 mg/gCr and 24.5 mg/gCr in people with FPG < 100 mg/dL and those with FPG ≥ 100 mg/dL, respectively. These were below the cutoff point for diagnosis of microalbuminuria (≥30 mg/gCr) [20]. The upper limit of normal UAE is considered as 10 mg/gCr assayed at a central laboratory (SRL, Inc., Tokyo, Japan), and UAE levels between 10 and 30 mg/gCr may primarily denote disturbance of albumin reabsorption in the proximal tubules without severe glomerular damage [21]. Festa et al. reported that urinary albumin-to-creatinine ratio was related to CRP, and that the association was consistent in nondiabetic people as well as those with type 2 diabetes. UAE and CRP are considered as markers of endothelial dysfunction and inflammation of the arterial wall, and they are related to insulin resistance [22,23]. Therefore, UAE was predictive of cardiovascular and non-cardiovascular mortality in the general population [2], even when slightly above the upper limit of normal, 10 mg/gCr [24]; hs-CRP also predicted all-cause mortality [25]. On the other hand, standard CRP was not significantly associated with FPG and fatty liver markers in our study. Plasma CRP concentrations increase in acute response to tissue inflammation and injury; however, hs-CRP is more accurate than standard CRP for measuring normal baseline concentrations, and is more useful for evaluating chronic inflammation. In atherosclerosis, which often has an inflammatory component [26], hs-CRP has been recognized to be a biomarker of atherosclerotic cardiovascular disease risk [27,28,29]. In our study, we did not detect a significant relationship between standard CRP and the cardiovascular risk markers examined, probably because CRP is an acute-phase reactant with a high intra-individual variability. Thus, standard CRP measurements in individuals might not accurately reflect the presence of subclinical low-grade inflammation. Hs-CRP is produced by the liver under conditions of inflammation, and independently increases in association with hepatic steatosis. Thus, hs-CRP might be more sensitive to basal and chronic low-grade inflammation [30]. Unfortunately, we could only evaluate hs-CRP in 175 people included in the subgroup analysis. Nevertheless, FLUS and TG in Period I were significantly and independently associated with hs-CRP in Period II in 120 people with the complete pairs of data. 

In our study ALT and other fatty liver markers in Period I correlated with FIRI, UAE, and hs-CRP in Period II. These results suggested that NAFLD contributes to insulin resistance and basal chronic low-grade inflammation in non-diabetic people, possibly increasing the risk of type 2 diabetes and cardiovascular events. Our results are in line with a recent Korean report of a cross-sectional study [31]. 

Some people, especially those with FPG ≥ 100 mg/dL, may have been undergone modifications of the covariates during the observation period. For example, glucose lowering, blood pressure lowering, and cholesterol lowering drug treatment might have started. A recent review stated that antidiabetic drugs seem to be promising drugs because they are able to treat both NAFLD manifestations and diabetes, preventing worsening of hepatic damage, but data are still conflicting [32]. Regretfully, information of prescriptions (generic name) were not included in our data. Nevertheless, the association between some fatty liver markers in Period II more clearly correlated with FIRI and UAE in Period II in the cross-sectional analyses (Table 3 and Table 5) compared with the longitudinal analyses (Table 2 and Table 4). 

There are several limitations that should be considered in our study. First, because we did not perform the oral glucose tolerance test (OGTT), some people with undiagnosed diabetes (i.e., those with FPG < 100 mg/dL but 2-h plasma glucose in an OGTT ≥200 mg/dL) might have been included in the lower FPG group. Second, we cannot assure that the participants were directly representative of the regional general population. Many participants were from the suburbs of Tokyo close to our University in the city center [33]. Third, we did not test other diabetes risk scores [34,35]; we decided to use the FINDRISC because it has been widely used and confirmed in many studies [5,36,37]. Fourth, although we carefully selected independent variables for regression analyses in consideration of multicollinearity, we could include only a few factors as independent variables, and unknown confounding factors may have contributed to our findings. Furthermore, our data were based on a single assessment at each timepoint, and this may have affected the accuracy of the measured values given a day-to-day variability. However, all measurements and samples were taken before breakfast and assays were performed in a single laboratory to minimize variability. Fifth, as we carried out retrospective analyses, we did not have measurements of all variables in all people, due to the contracts with people who participated in annual medical check-ups and their employers. Therefore, in linear regression analyses, numbers of complete pairs of datasets were smaller than numbers of evaluations of the variables, due to missing values. Finally, FIRI, UAE, CysC, and hs-CRP were not measured in Period I, and they were only evaluated in Period II as outcomes. 

## 5. Conclusions

In all people ALT, FLUS, and GGTP measured in Period I were independently associated with FIRI in Period II, whereas FPG was not. ALT was also associated with UAE measured in Period II in people with FPG < 100 mg/dL. Thus, some NAFLD markers may be usefully predictive of FIRI and UAE. The mechanism and cause/effect relationships should be elucidated by another prospective study in the future. 

## Figures and Tables

**Table 1:** Clinical profiles of 961 people for retrospective analysis.

Parameter (Reference Range and Unit)	All People	People with FPG < 100 mg/dL	People with FPG ≥ 100 mg/dL	*P* values between People with FPG < 100 and FPG ≥ 100 mg/dL
Number	961	778	183	NA
Gender (Men / Women)	613 / 348	471 / 307	142 / 41	<0.001 §
Period I				
Age (years)	43.5 (8.5)	42.5 (8.2)	47.3 (8.6)	<0.001
BMI (18.5–24.9 kg/m^2^)	22.8 (3.5)	22.5 (3.4)	24.3 (3.4)	<0.001
FPG (65–109 mg/dL)	94 (15)	89.9 (5.5)	112.7 (23.9)	<0.001
HbA1c (4.3–5.8%)	5.5 (0.6)	5.4 (0.3)	6.0 (1.0)	<0.001
Waist Circumference (cm)	82.1 (9.3)	81.0 (9.2)	86.5 (8.6)	<0.001
TG (30–149 mg/dL)	104 (81)	97 (70)	132 (115)	<0.001
HDL-C (40 ≤ mg/dL)	62 (16)	63 (16)	57 (15)	<0.001
TC (120–219 mg/dL)	203 (34)	201 (33)	212 (36)	<0.001
SBP (90–129 mmHg)	117 (12)	115 (12)	122 (13)	<0.001
DBP (50–84 mmHg)	74 (9)	73 (9)	78 (8)	<0.001
AST (10–30 U/L)	21 (8)	21 (8)	23 (10)	0.001
ALT (6–30 U/L)	23 (18)	22 (17)	29 (21)	<0.001
GGTP (15–50 U/L)	34 (33)	31 (27)	47 (47)	<0.001
ChE (200–450 U/L)	344 (71)	336 (72)	370 (64)	<0.001
ChE evaluations, *n*	744	581	163	<0.001 §
FLUS (points)	0.52 (0.72)	0.43 (0.65)	0.84 (0.86)	<0.001 #
FLUS evaluations (0/1/2 points, *n*)	461/189/100	386/149/51	75/40/49	0.317 ♮
Total JPDRISC (points)	4.0 (3.2)	3.7 (3.0)	5.7 (3.5)	<0.001
Standard CRP (mg/dL)	0.14 (0.49)	0.13 (0.50)	0.14 (0.43)	0.135 #
Standard CRP evaluations, *n*	463	378	85	0.613 §
Period II				
FIRI (μIU/mL)	6.7 (4.4)	6.2 (4.1)	8.6 (5.2)	<0.001
FIRI evaluations, *n*	961	778	183	0.177 §
UAE (mg/gCr)	14.0 (63.1)	11.5 (43.8)	24.5 (112.5)	<0.001 #
UAE evaluations, *n*	959	776	183	0.162 §
Serum cystatin C (mg/L)	0.64 (0.10)	0.63 (0.09)	0.68 (0.14)	0.011
Cystatin C evaluations, *n*	190	153	37	0.580 §
hs-CRP (ng/mL)	977 (2798)	663 (1173)	2190 (5620)	0.069 #
hs-CRP evaluations, *n*	175	139	36	0.353 §
Standard CRP (mg/dL)	0.08 (0.28)	0.08 (0.29)	0.09 (0.27)	0.319 #
Standard CRP evaluations, *n*	475	388	87	0.581 §

Values are means (SD). *P*–values were tested by unpaired-*t* test and *p* < 0.05 was considered significant. BMI—body mass index; FPG—fasting plasma glucose; HbA1c, glycated hemoglobin A1c; TG—serum triglycerides; HDL-C—serum high-density-lipoprotein cholesterol; TC—serum total cholesterol; SBP—systolic blood pressure; DBP—diastolic blood pressure; AST—serum aspartate aminotransferase; ALT—serum alanine aminotransferase; GGTP—serum gamma-glutamyltransferase; ChE—serum cholinesterase; FLUS—fatty liver using ultrasonography scores; JPDRISC—Japanese diabetes risk score; FIRI—serum fasting immunoreactive insulin; UAE—urinary albumin excretion; hs-CRP—serum high-sensitivity C-reactive protein; CRP—serum C-reactive protein; NA—not applicable. §—tested by chi-square test and *p* < 0.05 was considered significant. ♮—tested by Jonckheere–Terpstra test and *p* < 0.05 was considered significant. #—tested in logarithmic conversion.

**Table 2:** Linear regression coefficients (*β*) for the association between parameters measured in Period I and FIRI in Period II, in the total study population, and when stratified according to FPG.

	All People			People with FPG < 100 mg/dL			People with FPG ≥ 100 mg/dL		
Complete Pairs of Dataset, *n*	618			490			128		
Parameters	*β*	Standardized *β*	*P*	*β*	Standardized *β*	*P*	*β*	Standardized *β*	*P*
	(95% CIs of *β*)			(95% CIs of *β*)			(95% CIs of *β*)		
ALT (U/L)	0.084	0.327	<0.001	0.101	0.398	<0.001	0.052	0.206	0.016
	(0.064 to 0.104)			(0.078 to 0.124)			(0.010 to 0.093)		
FLUS (points)	1.182	0.187	<0.001	1.589	0.244	<0.001	0.403	0.068	0.441
	(0.694 to 1.671)			(1.039 to 2.139)			(−0.629 to 1.435)		
JPDRISC (points)	0.201	0.143	<0.001	0.151	0.109	0.005	0.380	0.257	0.001
	(0.106 to 0.297)			(0.046 to 0.255)			(0.153 to 0.608)		
ChE (U/L)	0.006	0.095	0.012	0.005	0.078	0.071	0.011	0.136	0.088
	(−0.001 to 0.011)			(0.000 to 0.009)			(−0.002 to 0.024)		
GGTP (U/L)	−0.011	−0.084	0.022	−0.023	−0.143	0.001	0.000	0.002	0.980
	(−0.020 to 0.002)			(−0.037 to −0.009)			(−0.014 to 0.014)		
TG (mg/dL)	0.009	0.169	<0.001	0.006	0.084	0.055	0.011	0.286	0.001
	(0.005 to 0.013)			(0.000 to 0.012)			(0.005 to 0.018)		
TC (mg/dL)	−0.001	−0.010	0.769	−0.002	−0.016	0.672	0.005	0.033	0.665
	(−0.010 to 0.007)			(−0.011 to 0.007)			(−0.017 to 0.026)		
DBP (mmHg)	0.017	0.034	0.317	0.011	0.023	0.554	0.035	0.056	0.430
	(−0.017 to 0.051)			(−0.025 to 0.047)			(−0.053 to 0.123)		
FPG (mg/dL)	0.014	0.048	0.163	0.074	0.096	0.010	−0.003	−0.016	0.833
	(−0.005 to 0.033)			(0.018 to 0.131)			(−0.032 to 0.026)		
Constant	−1.782	NA	0.248	−5.822	NA	0.032	−4.618	NA	0.267
	(−4.806 to 1.242)			(−11.148 to −0.495)			(−12.819 to 3.583)		

FIRI–fasting immunoreactive insulin; *β*—regression coefficient; CIs—confidence intervals; ALT—serum alanine aminotransferase; FLUS—fatty liver using ultrasonography scores; JPDRISC—Japanese diabetes risk score; ChE—serum cholinesterase; GGTP—serum gamma-glutamyltransferase; TG—serum triglycerides; TC—serum total cholesterol; DBP—diastolic blood pressure; FPG—fasting plasma glucose; NA—not applicable. Factors of age and body mass index were included into JPDRISC.

**Table 3:** Linear regression coefficients (*β*) for the association between parameters measured in Period II and FIRI in Period II, in the total study population, and when stratified according to FPG.

	All People			People with FPG < 100 mg/dL			People with FPG ≥ 100 mg/dL		
Complete Pairs of Dataset, *n*	873			686			187		
Parameters	*β*	Standardized *β*	*P*	*β*	Standardized *β*	*P*	*β*	Standardized *β*	*P*
	(95% CIs of *β*)			(95% CIs of *β*)			(95% CIs of *β*)		
ALT (U/L)	0.070	0.287	<0.001	0.067	0.262	<0.001	0.074	0.338	<0.001
	(0.054 to 0.086)			(0.048 to 0.086)			(0.043 to 0.105)		
FLUS (points)	1.558	0.261	<0.001	1.577	0.272	<0.001	1.484	0.236	0.001
	(1.178 to 1.938)			(1.167 to 1.988)			(−0.618 to 2.349)		
JPDRISC (points)	−0.001	−0.010	0.693	0.002	0.035	0.240	−0.003	−0.058	0.298
	(0.004 to 0.003)			(−0.002 to 0.007)			(−0.009 to 0.003)		
ChE (U/L)	0.007	0.116	<0.001	0.003	0.063	0.071	0.014	0.180	0.006
	(−0.003 to 0.010)			(0.000 to 0.007)			(−0.004 to 0.024)		
GGTP (U/L)	−0.009	−0.074	0.012	−0.013	−0.121	<0.001	0.004	0.027	0.661
	(−0.016 to −0.002)			(−0.021 to −0.006)			(−0.013 to 0.020)		
TG (mg/dL)	0.009	0.151	<0.001	0.010	0.173	<0.001	0.005	0.097	0.161
	(0.005 to 0.012)			(0.006 to 0.015)			(−0.002 to 0.012)		
TC (mg/dL)	0.002	0.018	0.511	−0.003	−0.021	0.487	0.008	0.055	0.380
	(−0.005 to 0.009)			(−0.010 to 0.005)			(−0.010 to 0.026)		
DBP (mmHg)	0.041	0.085	0.003	0.035	0.083	0.010	0.034	0.051	0.391
	(0.014 to 0.068)			(0.009 to 0.062)			(−0.044 to 0.113)		
FPG (mg/dL)	0.017	0.065	0.021	0.137	0.203	<0.001	−0.010	−0.051	0.414
	(0.002 to 0.031)			(0.096 to 0.177)			(−0.036 to 0.015)		
Constant	−3.686	NA	0.002	−11.900	NA	<0.001	−3.978	NA	0.257
	(−6.058 to −1.314)			(−15.666 to −8.134)			(−10.882 to 2.926)		

FIRI—fasting immunoreactive insulin; *β*—regression coefficient; CIs—confidence intervals; ALT—serum alanine aminotransferase; FLUS—fatty liver using ultrasonography scores; JPDRISC—Japanese diabetes risk score; ChE—serum cholinesterase; GGTP—serum gamma-glutamyltransferase; TG—serum triglycerides; TC—serum total cholesterol; DBP—diastolic blood pressure; FPG—fasting plasma glucose; NA—not applicable. Factors of age and body mass index were included into JPDRISC.

**Table 4:** Linear regression coefficients (*β*) for the association between parameters measured in Period I and UAE in Period II, in the total study population, and when stratified according to FPG.

	All People			People with FPG < 100 mg/dL			People with FPG ≥ 100 mg/dL		
Complete Pairs of Dataset, *n*	616			488			128		
Parameters	*β*	Standardized *β*	*P*	*β*	Standardized *β*	*P*	*β*	Standardized *β*	*P*
	(95% CIs of *β*)			(95% CIs of *β*)			(95% CIs of *β*)		
ALT (U/L)	0.323	0.084	0.069	0.485	0.263	<0.001	0.158	0.024	0.798
	(−0.026 to 0.672)			(0.279 to 0.692)			(−1.062 to 1.379)		
FLUS (points)	−2.213	−0.023	0.611	0.549	0.012	0.827	−12.993	−0.083	0.395
	(−10.761 to 6.334)			(−4.379 to 5.477)			(−43.160 to 17.173)		
JPDRISC (points)	−0.857	−0.041	0.314	0.243	0.024	0.610	−1.922	−0.049	0.568
	(−2.527 to 0.813)			(−0.692 to 1.178)			(−8.566 to 4.722)		
ChE (U/L)	−0.033	−0.035	0.426	0.012	0.028	0.594	−0.185	−0.086	0.326
	(−0.114 to 0.048)			(−0.032 to 0.056)			(−0.556 to 0.186)		
GGTP (U/L)	−0.234	−0.122	0.005	−0.119	−0.105	0.061	−0.345	−0.140	0.097
	(−0.396 to −0.072)			(−0.244 to 0.005)			(−0.754 to 0.063)		
TG (mg/dL)	0.036	0.043	0.321	0.005	0.009	0.867	0.149	0.142	0.120
	(−0.035 to 0.107)			(−0.049 to 0.058)			(−0.039 to 0.337)		
TC (mg/dL)	−0.159	−0.078	0.049	−0.016	−0.017	0.726	−0.703	−0.188	0.028
	(−0.318 to −0.001)			(−0.103 to 0.072)			(−1.331 to −0.075)		
DBP (mmHg)	0.320	0.043	0.290	0.231	0.068	0.162	1.072	0.065	0.411
	(−0.273 to 0.914)			(−0.094 to 0.556)			(−1.498 to 3.641)		
FPG (mg/dL)	1.907	0.453	<0.001	0.071	0.013	0.782	3.018	0.591	<0.001
	(1.574 to 2.240)			(−0.435 to 0.578)			(2.171 to 3.865)		
Constant	−143.820	NA	<0.001	−21.505	NA	0.380	−162.343	NA	0.182
	(−0.318 to −0.001)			(−69.602 to 26.593)			(−402.039 to 77.353)		

UAE—urinary albumin excretion; *β*—regression coefficient; CIs—confidence intervals; ALT—alanine aminotransferase; FLUS—fatty liver using ultrasonography scores; JPDRISC—Japanese diabetes risk score; ChE—cholinesterase; GGTP—gamma-glutamyltransferase; TG—triglyceride; TC—total cholesterol; DBP—diastolic blood pressure; FPG—fasting plasma glucose; NA—not applicable. Factors of age and body mass index were included into JPDRISC.

**Table 5:** Linear regression coefficients (*β*) for the association between parameters measured in Period II and UAE in Period II, in the total study population, and when stratified according to FPG.

	All People			People with FPG < 100 mg/dL			People with FPG ≥ 100 mg/dL		
Complete Pairs of Dataset, *n*	872			685			187		
Parameters	*β*	Standardized *β*	*P*	*β*	Standardized *β*	*P*	*β*	Standardized *β*	*P*
	(95% CIs of *β*)			(95% CIs of *β*)			(95% CIs of *β*)		
ALT (U/L)	−0.600	−0.106	0.010	0.118	0.068	0.172	−1.716	−0.205	0.016
	(−1.054 to 0.146)			(−0.052 to 0.288)			(−3.110 to −0.322)		
FLUS (points)	−2.759	−0.020	0.618	3.139	0.080	0.092	-9.932	−0.041	0.615
	(−13.621 to 8.104)			(−0.518 to 6.795)			(−48.784 to 28.920)		
JPDRISC (points)	−0.010	−0.007	0.833	−0.006	−0.013	0.734	−0.029	−0.014	0.828
	(−0.101 to 0.082)			(−0.043 to 0.030)			(−0.289 to 0.232)		
ChE (U/L)	−0.089	−0.065	0.082	−0.011	−0.030	0.510	−0.306	−0.103	0.182
	(−0.190 to 0.012)			(−0.042 to 0.021)			(−0.755 to 0.114)		
GGTP (U/L)	−0.045	−0.016	0.656	−0.030	−0.040	0.373	−0.153	−0.030	0.680
	(−0.242 to −0.152)			(−0.096 to 0.036)			(−0.884 to 0.577)		
TG (mg/dL)	0.081	0.061	0.119	−0.015	−0.037	0.432	0.198	0.099	0.223
	(−0.021 to 0.183)			(−0.052 to 0.022)			(−0.121 to 0.517)		
TC (mg/dL)	0.234	0.076	0.024	0.061	0.074	0.064	0.957	0.169	0.022
	(0.031 to 0.436)			(−0.004 to 0.126)			(0.137 to 1.776)		
DBP (mmHg)	0.115	0.010	0.768	0.330	0.113	0.007	0.379	0.015	0.832
	(−0.650 to 0.880)			(−0.092 to 0.568)			(−3.138 to 3.897)		
FPG (mg/dL)	2.435	0.406	<0.001	−0.083	−0.018	0.651	3.896	0.495	<0.001
	(2.030 to 2.840)			(−0.442 to 0.276)			(2.767 to 5.025)		
Constant	−230.442	NA	<0.001	−16.308	NA	0.339	−476.193	NA	0.003
	(−298.134 to −162.751)			(−49.776 to 17.160)			(−786.094 to −166.293)		

UAE—urinary albumin excretion; *β*—regression coefficient; CIs—confidence intervals; ALT—alanine aminotransferase; FLUS—fatty liver using ultrasonography scores; JPDRISC—Japanese diabetes risk score; ChE—cholinesterase; GGTP—gamma-glutamyltransferase; TG—triglyceride; TC—total cholesterol; DBP—diastolic blood pressure; FPG—fasting plasma glucose; NA—not applicable. Factors of age and body mass index were included into JPDRISC.

## Supplementary Materials

The following are available online at https://www.mdpi.com/2077-0383/9/10/3161/s1, Table S1: The study-specific Japanese Diabetes Risk Score (JPDRISC) and the original Finnish Diabetes Risk Score (FINDRISC) underlined; Table S2: Clinical profiles of 961 people for retrospective analysis; skewness and kurtosis of variables; Table S3: Spearman’s rank-order correlation between FIRI in Period II and variables in Period I; Table S4: Spearman’s rank-order correlation between UAE in Period II and variables in Period I; Table S5: Spearman’s rank-order correlation between hs-CRP in Period II and variables in Period I; Table S6: Linear regression coefficients (β) for the association between parameters measured in Period I and high-sensitivity C-reactive protein in Period II; Table S7: Linear regression coefficients (β) for the association between parameters measured in Period II and high-sensitivity C-reactive protein in Period II; Figure S1: Flow chart of the study population selection. Number of people recruited and excluded, and the reason of the exclusion; Figure S2: Histogram of variables in all people in Period I; Figure S3: Histogram of variables in all people in Period I; Figure S4: Histogram of variables in all people in Period I; Figure S5: Histogram of variables in all people in Period II.

## References

[1] The DECODE Insulin Study Group (2004). Plasma insulin and cardiovascular mortality in non-diabetic European men and women: A meta-analysis of data from eleven prospective studies. Diabetologia.

[2] Hillege H.L., Fidler V., Diercks G.F., van Gilst W.H., de Zeeuw D., van Veldhuisen D.J., Gans R.O.B., Janssen W.M.T., Grobbee D.E., de Jong P.E. (2002). Urinary albumin excretion predicts cardiovascular and noncardiovascular mortality in general population. Circulation.

[3] Ratziu V., Bellentani S., Cortez-Pinto H., Day C., Marchesini G. (2010). A position statement on NAFLD/NASH based on the EASL 2009 special conference. J. Hepatol..

[4] Katoh S., Peltonen M., Wada T., Zeniya M., Sakamoto Y., Utsunomiya K., Tuomilehto J. (2014). Fatty liver and serum cholinesterase are independently correlated with HbA1c levels: Cross-sectional analysis of 5384 people. J. Int. Med. Res..

[5] Katoh S., Peltonen M., Zeniya M., Kaji M., Sakamoto Y., Utsunomiya K., Tuomilehto J. (2016). Analysis of the Japanese Diabetes Risk Score and fatty liver markers for incident diabetes in a Japanese cohort. Prim. Care Diabetes.

[6] Eisenberg R.L. (2003). Clinical Imaging: An Atlas of Differential Diagnosis.

[7] Kunz E., Kuntz H.D. (2002). Hepatology Principles and Practice.

[8] Boyer T.D., Manns M.P., Sanyal A.J. (2012). Zakin and Boyer’s Hepatology.

[9] Osawa H., Mori Y. (1996). Sonographic diagnosis of fatty liver using a histogram technique that compares liver and renal cortical echo amplitudes. J. Clin. Ultrasound.

[10] Lindström J., Tuomilehto J. (2003). The diabetes risk score: A practical tool to predict type 2 diabetes risk. Diabetes Care.

[11] International Diabetes Federation (2016). IDF Consensus Worldwide Definition of the Metabolic Syndrome. https://www.idf.org/e-library/consensus-statements/60-idfconsensus-worldwide-definitionof-the-metabolic-syndrome.html.

[12] NGSP Steering Committee (2016). NGSP Protocol. http://www.ngsp.org/protocol.asp.

[13] American Diabetes Association (2020). 2. Classification and diagnosis of diabetes: Standards of medical care in diabetes—2020. Diabetes Care.

[14] Kong A.P., Choi K.C., Cockram C.S., Ho C.S., Chan M.H., Ozaki R., Wong G.W.K., Ko G.T.C., So W.Y., Tong P.C.Y. (2008). Independent associations of alanine aminotransferase (ALT) levels with cardiovascular risk factor clustering in Chinese adolescents. J. Hepatol..

[15] Bril F., Lomonaco R., Orsak B., Ortiz-Lopez C., Webb A., Tio F., Hecht J., Cusi K. (2014). Relationship between disease severity, hyperinsulinemia, and impaired insulin clearance in patients with nonalcoholic steatohepatitis. Hepatology.

[16] Ford E.S., Schulze M.B., Bergmann M.M., Thamer C., Joost H.G., Boeing H. (2008). Liver enzymes and incident diabetes: Findings from the European Prospective Investigation into Cancer and Nutrition (EPIC)-Potsdam Study. Diabetes Care.

[17] Kim H.C., Nam C.M., Jee S.H., Han K.H., Oh D.K., Suh I. (2004). Normal serum aminotransferase concentration and risk of mortality from liver diseases: Prospective cohort study. BMJ.

[18] Bae J.C., Cho Y.K., Lee W.Y., Seo H.I., Rhee E.J., Park S.E., Park C.Y., Oh K.W., Sung K.C., Kim B.I. (2010). Impact of nonalcoholic fatty liver disease on insulin resistance in relation to HbA1c levels in nondiabetic subjects. Am. J. Gastroenterol..

[19] Hanley A.J., Williams K., Festa A., Wagenknecht L.E., D’Agostino R.B., Haffner S.M. (2005). Liver markers and development of the metabolic syndrome: The insulin resistance atherosclerosis study. Diabetes.

[20] Roshan B., Stanton R.C. (2013). A story of microalbuminuria and diabetic nephropathy. J. Nephropathol..

[21] Tojo A., Onozato M.L., Kurihara H., Sakai T., Goto A., Fujita T. (2003). Angiotensin II blockade restores albumin reabsorption in the proximal tubules of diabetic rats. Hypertens. Res..

[22] Pannacciulli N., Cantatore F.P., Minenna A., Bellacicco M., Giorgino R., De Pergola G. (2001). Urinary albumin excretion is independently associated with C-reactive protein levels in overweight and obese nondiabetic premenopausal women. J. Intern. Med..

[23] Lee I.T., Lee W.J., Huang C.N., Sheu W.H.-H. (2007). The association of low-grade inflammation, urinary albumin, and insulin resistance with metabolic syndrome in nondiabetic Taiwanese. Metabolism.

[24] Matsushita K., van der Velde M., Astor B.C., Woodward M., Levey A.S., de Jong P.E., Coresh J., Gansevoort R.T., Chronic Kidney Disease Prognosis Consortium (2010). Association of estimated glomerular filtration rate and albuminuria with all-cause and cardiovascular mortality in general population cohorts: A collaborative meta-analysis. Lancet.

[25] Ridker P.M. (2008). High-sensitivity C-reactive protein as a predictor of all-cause mortality: Implications for research and patient care. Clin. Chem..

[26] Ridker P.M., Everett B.M., Thuren T., MacFadyen J.G., Chang W.H., Ballantyne C., Fonseca F., Nicolau J., Koenig W., Anker S.D. (2017). Antiinflammatory therapy with canakinumab for atherosclerotic disease. N. Engl. J. Med..

[27] Reiner Ž., Catapano A.L., De Backer G., Graham I., Taskinen M.R., Wiklund O., Agewall S., Alegria E., Chapman M.J., Durrington P. (2011). ESC/EAS Guidelines for the management of dyslipidaemias: The Task Force for the management of dyslipidaemias of the European Society of Cardiology (ESC) and the European Atherosclerosis Society (EAS). Eur. Heart J..

[28] Goff D.C., Lloyd-Jones D.M., Bennett G., Coady S., D’Agostino R.B., Gibbons R., Greenland P., Lackland D.T., Levy D., O’Donnell C.J. (2014). 2013 ACC/AHA Guideline on the assessment of cardiovascular risk. Circulation.

[29] Jacobson T.A., Ito M.K., Maki K.C., Orringer C.E., Bays H.E., Jones P.H., McKenney J.M., Grundy S.M., Gill E.A., Wild R.A. (2015). National lipid association recommendations for patient-centered management of dyslipidemia: Part 1—Full report. J. Clin. Lipidol..

[30] Luyendyk J.P., Guo G.L. (2011). Steatosis DeLIVERs high-sensitivity C-reactive protein. Arterioscler. Thromb. Vasc. Biol..

[31] Choi J.W., Oh I.H., Lee C.H., Park J.S. (2018). Is there a J-shaped relationship between the fatty liver index and risk of microalbuminuria in the general population?. Clin. Chim. Acta.

[32] Tacelli M., Celsa C., Magro B., Giannetti A., Pennisi G., Spatola F., Petta S. (2018). Antidiabetic drugs in NAFLD: The accomplishment of two goals at once?. Pharmaceuticals.

[33] Wada T., Urashima M., Fukumoto T. (2007). Risk of metabolic syndrome persists twenty years after the cessation of smoking. Intern. Med..

[34] Mühlenbruch K., Ludwig T., Jeppesen C., Joost H.G., Rathmann W., Meisinger C., Peters A., Boeing H., Thorand B., Schulze M.B. (2014). Update of the German Diabetes Risk Score and external validation in the German MONICA/KORA study. Diabetes Res. Clin. Pract..

[35] Gray L.J., Khunti K., Wilmot E.G., Yates T., Davies M.J. (2014). External validation of two diabetes risk scores in a young UK South Asian population. Diabetes Res. Clin. Pract..

[36] Alssema M., Vistisen D., Heymans M.W., Nijpels G., Glümer C., Zimmet P.Z., Shaw J.E., Eliasson M., Stehouwer C.D.A., Tabák A.G. (2011). The evaluation of screening and early detection strategies for type 2 diabetes and impaired glucose tolerance (DETECT-2) update of the Finnish Diabetes Risk Score for prediction of incident type 2 diabetes. Diabetologia.

[37] Tankova T., Chakarova N., Atanassova I., Dakovska L. (2011). Evaluation of the Finnish Diabetes Risk Score as a screening tool for impaired fasting glucose, impaired glucose tolerance and undetected diabetes. Diabetes Res. Clin. Pract..

