# Supplementary material for: Non-Alcoholic Fatty Liver Disease Markers Associated with Fasting Serum Insulin and Urinary Albumin Excretion Independent of Fasting Plasma Glucose
Source: J Clin Med. 2020 Sep 29;9(10):3161. doi: 10.3390/jcm9103161 (PMC7650561; doi:10.3390/jcm9103161)
Supplement: Supplementary file 1 [file jcm-09-03161-s001.zip › jcm-925882-supplementary.docx]

**
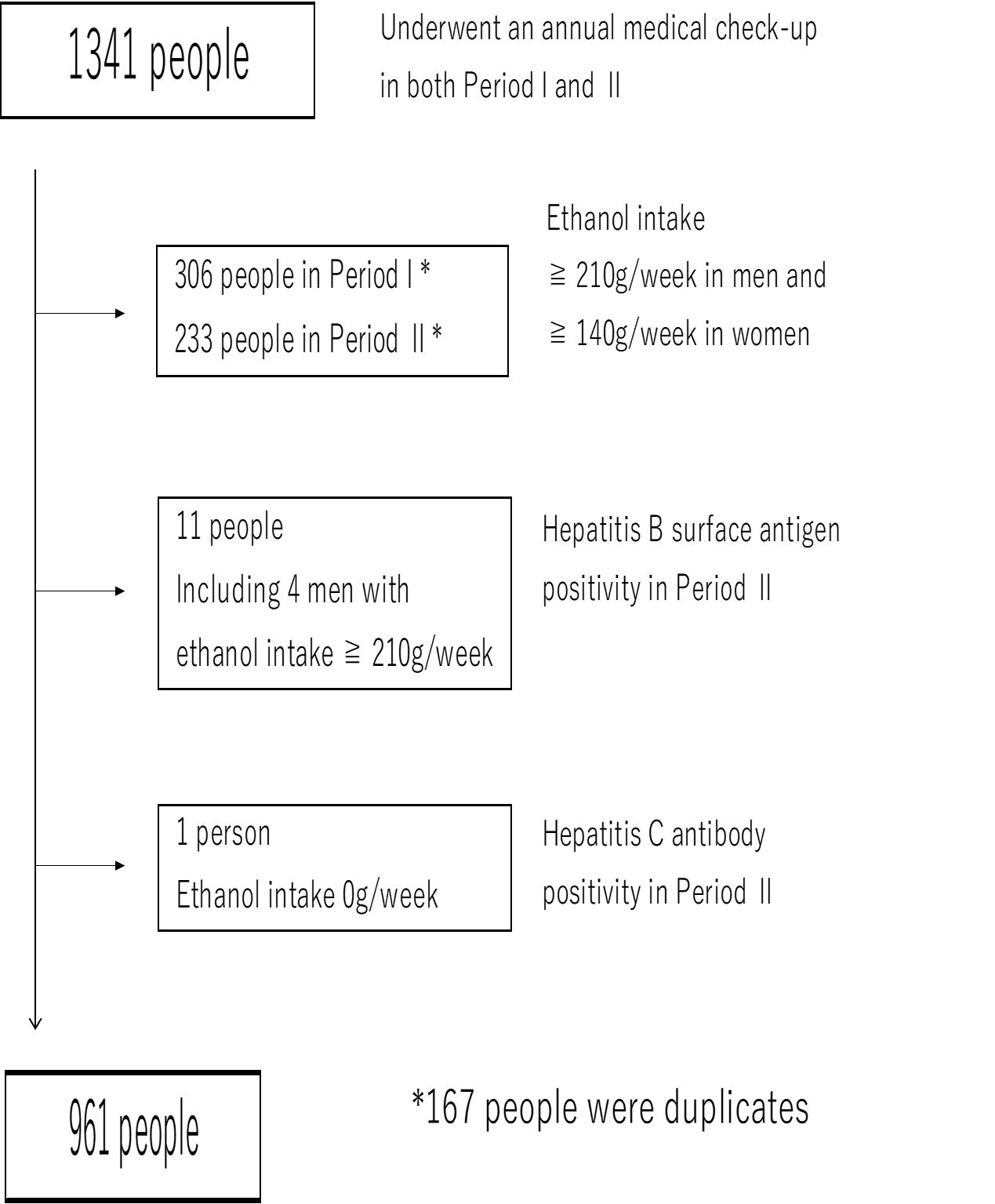
**

**Supplemental Figure S1.** Flow chart of the study population selection. Number of people recruited and excluded, and the reason of the exclusion.

**Supplemental Table S1:** Study-specific Japanese Diabetes Risk Score (JPDRISC)　and original Finnish Diabetes Risk Score (FINDRISC) underlined.

**1. Age** Under 45 years (0 p.)
 45–54 years (2 p.)
 55–64 years (3 p.)
 Over 64 years (4 p.)

**2. Body-mass index**

Lower than 25 kg/m^2^ (0 p.)

25 - 30 kg/m^2^ (1 p.)

30 kg/m^2^ or higher (3 p.)

**3. Waist circumference measured below the ribs (usually at the level of the navel)** MEN WOMEN

Less than 90 cm (94cm) Less than 80 cm (0 p.)

90-102 cm 80 – 88 cm (3 p.)

102 cm or more 88 cm or more (4 p.)

**4. Do you usually have daily at least 30 minutes of physical activity at work and/or during leisure time (including normal daily activity)?**
 Yes (0 p.)
 No (2 p.)

**5. How often do you eat vegetables, fruit or berries?** Every day (0 p.)
 Not every day (1 p.)

**6. Have you ever taken antihypertensive medication regularly?**

No (0 p.)
 Yes (2 p.)

**7. Have you ever been found to have high blood glucose (eg in a health examination, during an illness, during pregnancy)?**

No (0 p.)
 Yes (5 p.)

**8. Have any of the members of your immediate family or other relatives been diagnosed with diabetes (type 1 or type 2)?**

No (0 p.)

Yes: grandparent, aunt, uncle or first cousin (but no own parent, brother, sister or child) (3 p.)

Yes: parent, brother, sister or own child (5 p.)


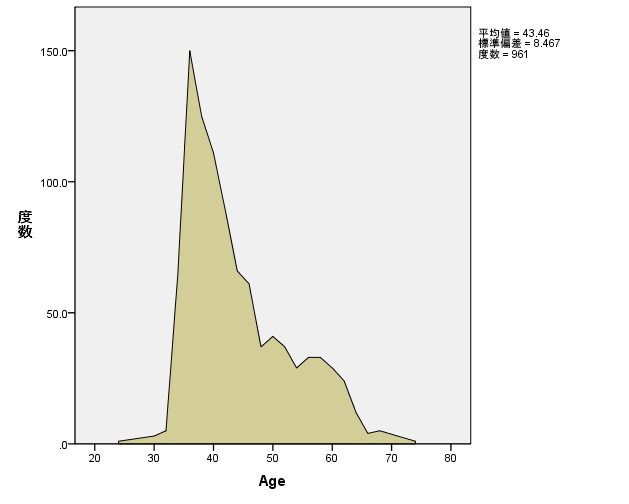

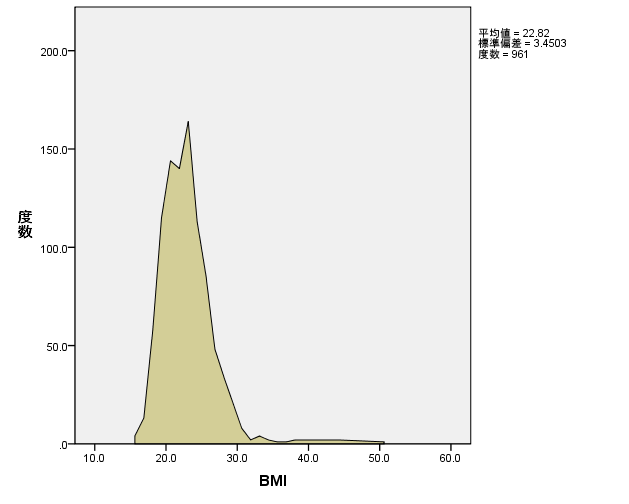

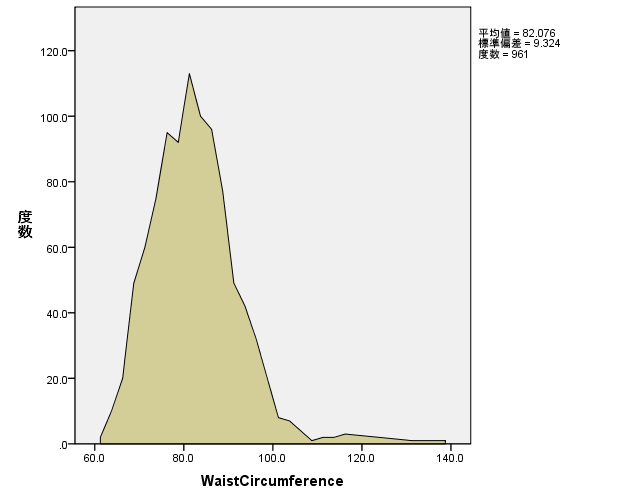

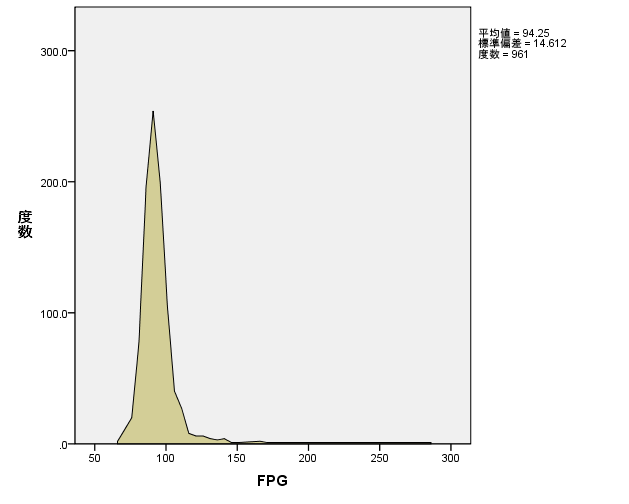

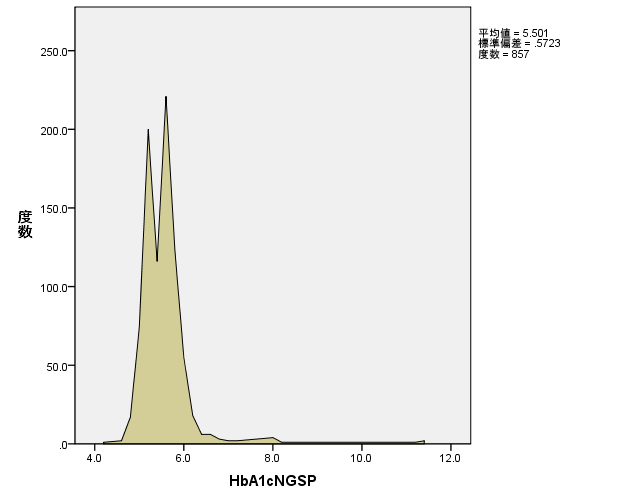

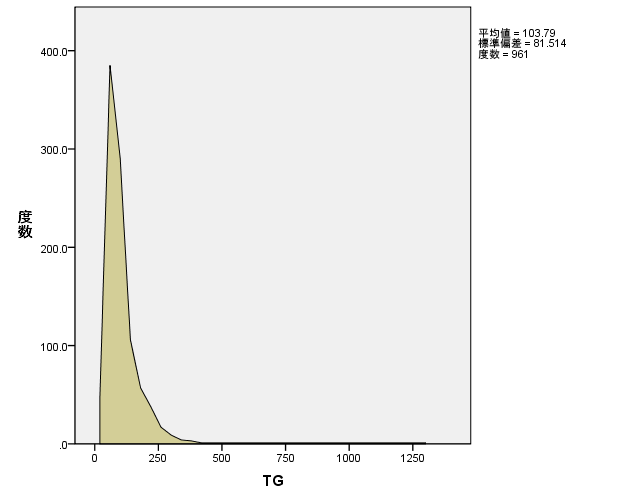

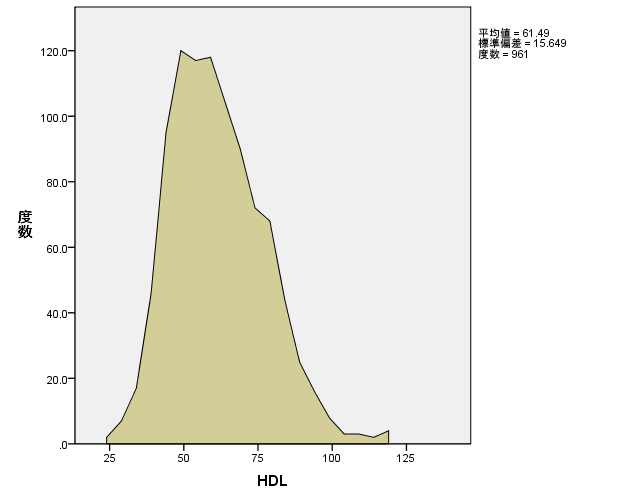

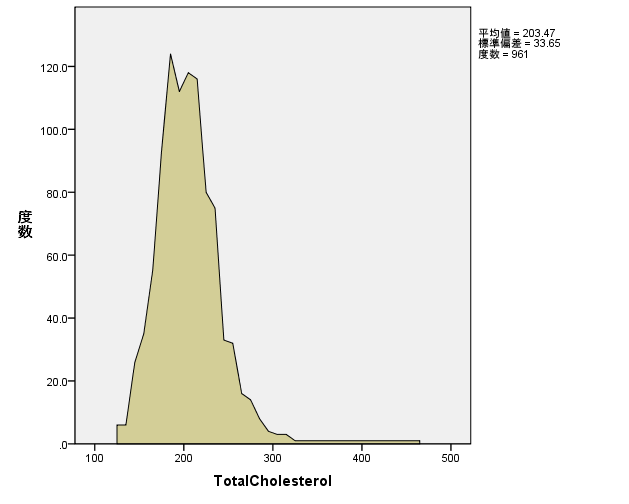


**Supplemental Figure S2**. Histogram of variables in all people in Period I. Y-axis: number of people, X-axis: a range of values.


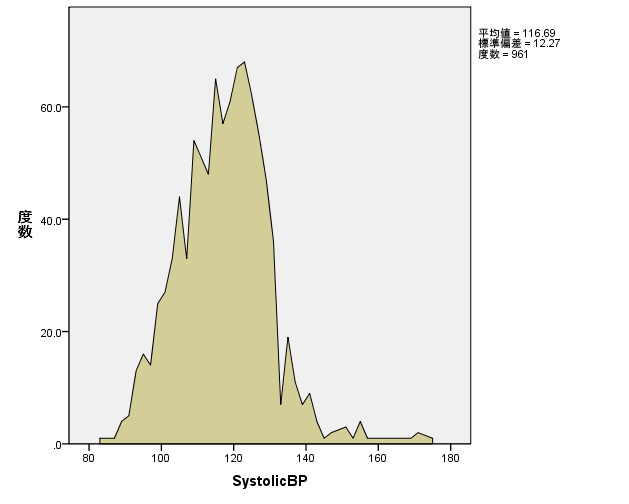

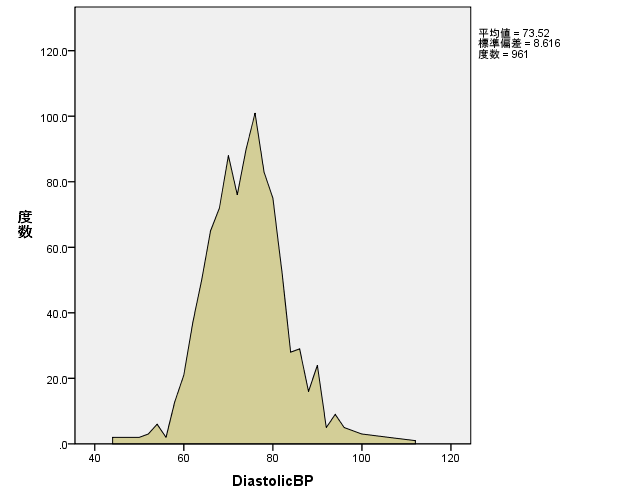

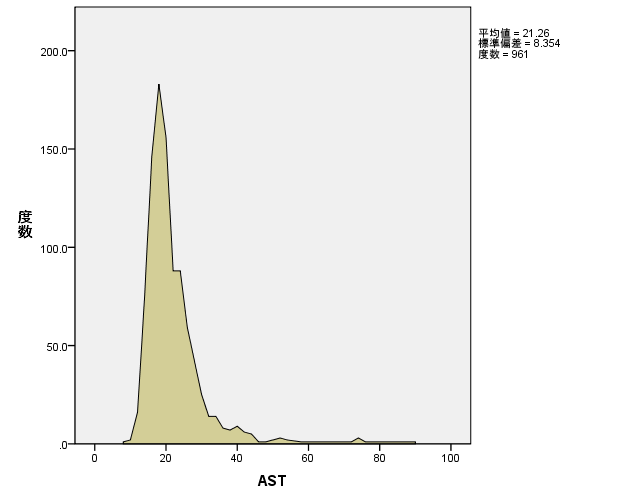

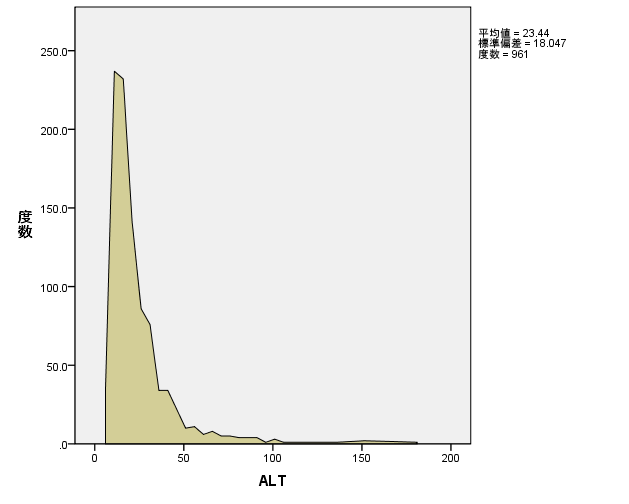

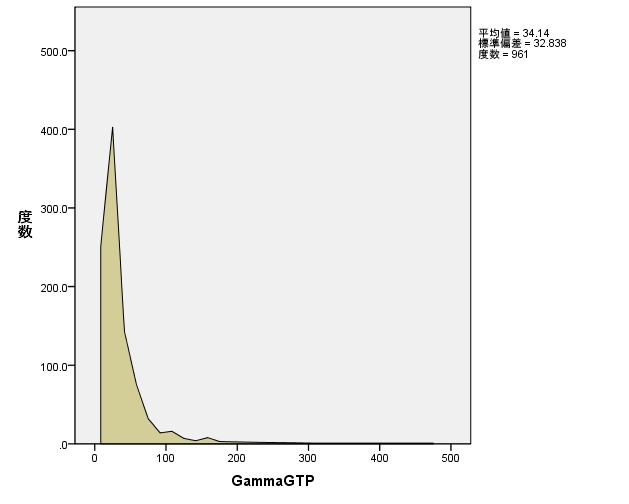

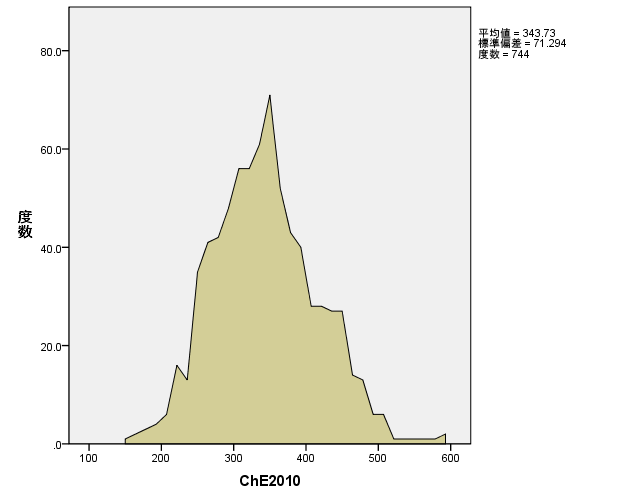


Cholinesterase


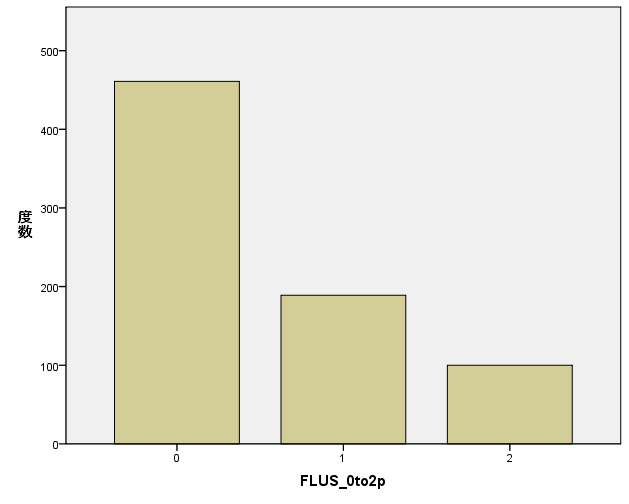


Mild

Normal

Moderate or severe


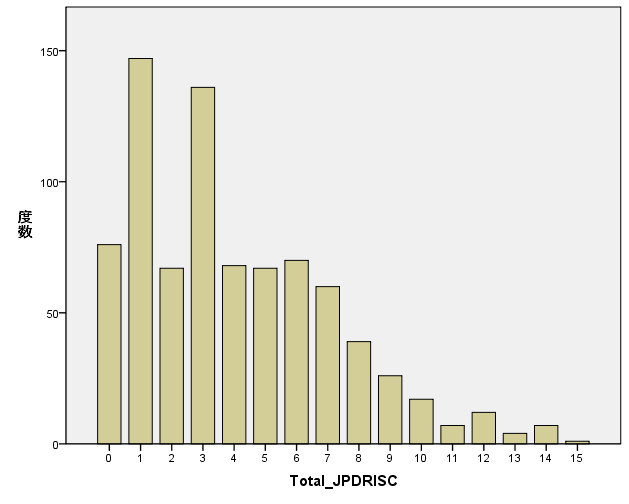


Low risk High Risk

0 1 2

Fatty liver using ultrasonography score (points)

Total Japanese Diabetes Risk Score (points)

**Supplemental Figure S3.** Histogram of variables in all people in Period I. Y-axis: number of people, X-axis: a range of values.


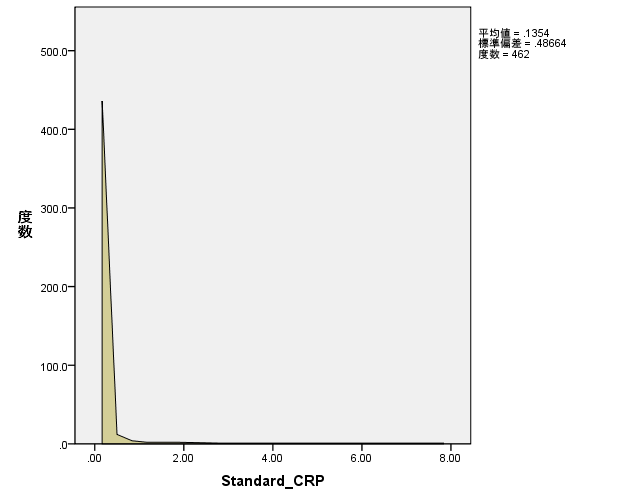


Standard CRP

**Supplemental Figure S4.** Histogram of variables in all people in Period I. Y-axis: number of people, X-axis: a range of values.

**
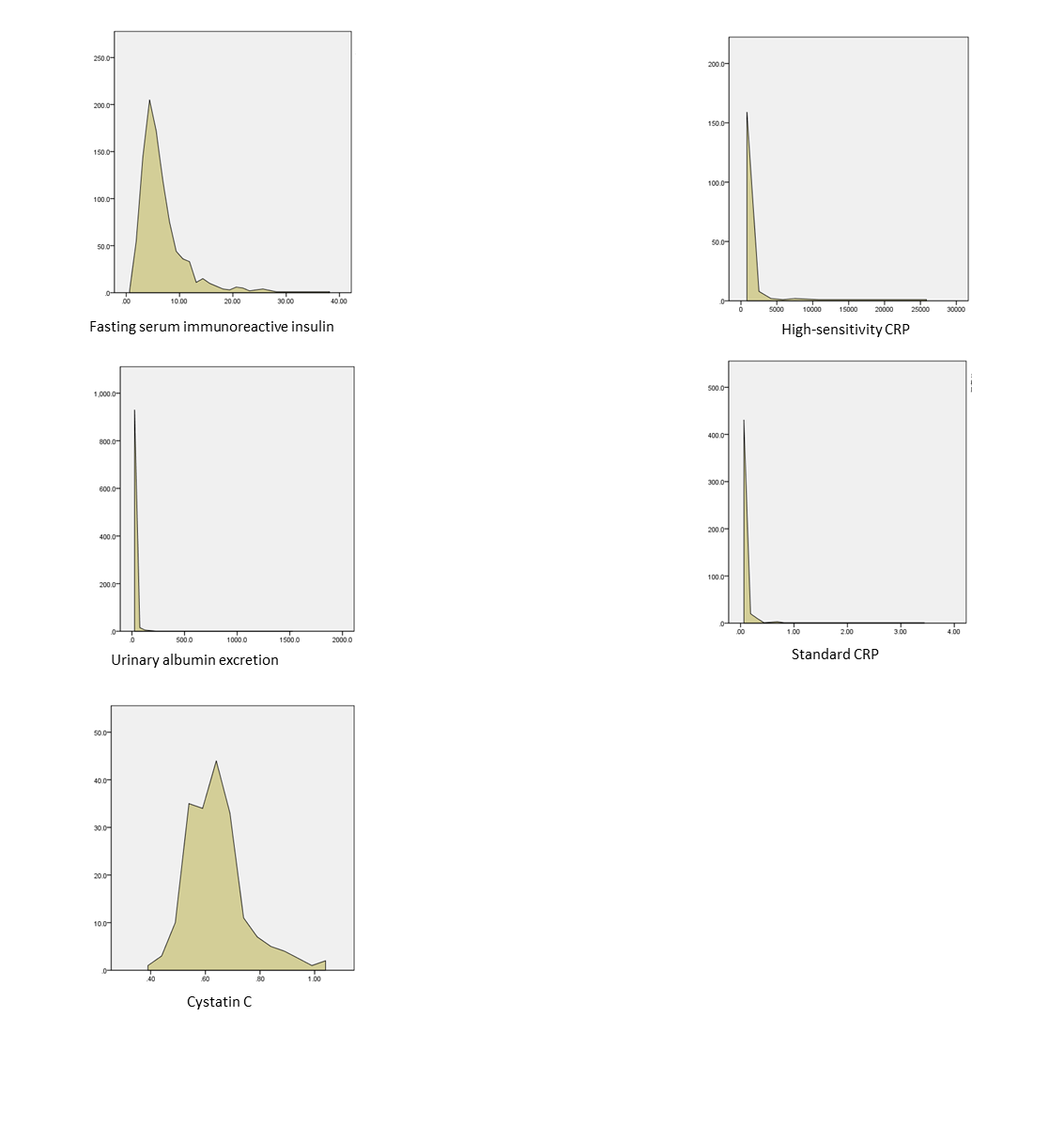
**

**Supplemental Figure S5.** Histogram of variables in all people in Period II. Y-axis: number of people, X-axis: a range of values.

**Supplemental Table S2.** Clinical profiles of 961 people for retrospective analysis; skewness and kurtosis of variables.

| Parameter　(Reference Range and Unit) | All people | | People with FPG<100 mg/dl | | People with FPG≥100 mg/dl | |
| --- | --- | --- | --- | --- | --- | --- |
|  | Skewness | Kurtosis | Skewness | Kurtosis | Skewness | Kurtosis |
|  |  |  |  |  |  |  |
| Period I |  |  |  |  |  |  |
|  |  |  |  |  |  |  |
| Age (years) | 0.869 (0.079) | -0.152 (0.158) | 1.081 (0.088) | 0.418 (0.175) | 0.203 (0.180) | -1.001 (0.357) |
|  |  |  |  |  |  |  |
| BMI (18.5-24.9kg/m^2^) | 1.618 (0.079) | 7.556 (0.158) | 1.915 (0.08) | 10.323 (0.175) | 0.896 (0.180) | 1.621 (0.357) |
|  |  |  |  |  |  |  |
| FPG (65-109mg/dL) | 5.649 (0.079) | 53.672 (0.158) | -0.477 (0.088) | 0.092 (0.175) | 4.231 (0.180) | 22.265 (0.357) |
|  |  |  |  |  |  |  |
| HbA1c (4.3-5.8%) | 5.022 (0.084) | 42.333 (0.167) | -0.041 (0.094) | -0.212 (0.187) | 3.455 (0.184) | 14.471 (0.366) |
|  |  |  |  |  |  |  |
| Waist Circumference (cm) | 0.781 (0.079) | 2.195 (0.158) | 0.919 (0.088) | 3.019 (0.175) | 0.566 (0.180) | 0.504 (0.357) |
|  |  |  |  |  |  |  |
| TG (30-149mg/dL) | 6.719 (0.079) | 81.709 (0.158) | 6.135 (0.088) | 76.098 (0.175) | 6.371 (0.180) | 60.858 (0.357) |
|  |  |  |  |  |  |  |
| HDL-C (40≤ mg/dL) | 0.591 (0.079) | 0.373 (0.158) | 0.544 (0.088) | 0.174 (0.175) | 0.879 (0.180) | 1.869 (0.357) |
|  |  |  |  |  |  |  |
| TC (120-219mg/dL) | 0.889 (0.079) | 3.947 (0.158) | 1.007 (0.088) | 5.225 (0.175) | 0.459 (0.180) | 0.642 (0.357) |
|  |  |  |  |  |  |  |
| SBP (90-129mmHg) | 0.409 (0.079) | 1.349 (0.158) | 0.203 (0.088) | 0.629 (0.175) | 0.805 (0.180) | 2.402 (0.357) |
|  |  |  |  |  |  |  |
| DBP (50-84mmHg) | 0.262 (0.079) | 0.765 (0.158) | 0.247 (0.088) | 0.791 (0.175) | 0.555 (0.180) | 0.837 (0.357) |
|  |  |  |  |  |  |  |
| AST (10-30U/L) | 3.317 (0.079) | 17.486 (0.158) | 3.224 (0.088) | 17.680 (0.175) | 3.187 (0.180) | 13.811 (0.357) |
|  |  |  |  |  |  |  |
| ALT (6-30U/L) | 3.336 (0.079) | 16.754 (0.158) | 3.582 (0.088) | 20.312 (0.175) | 2.719 (0.180) | 93686 (0.357) |
|  |  |  |  |  |  |  |
| GGTP (15-50U/L) | 4.868 (0.079) | 44.267 (0.158) | 3.634 (0.088) | 20.813 (0.175) | 4.991 (0.180) | 38.844 (0.357) |
|  |  |  |  |  |  |  |
| ChE (200-450U/L) | 0.337 (0.090) | 0.060 (0.179) | 0.463 (0.101) | 0.358 (0.202) | 0.080 (0.190) | -0.561 (0.378) |
|  |  |  |  |  |  |  |
| FLUS (points) | 1.015 (0.089) | -0.369 (0.178) | 1.234 (0.101) | 0.320 (0.202) | 0.312 (0.190) | -1.574 (0.377) |
|  |  |  |  |  |  |  |
| Total JPDRISC (points) | 0.850 (0.086) | 0.316 (0.172) | 0.908 (0.095) | 0.471 (0.190) | 0.494 (0.201) | -0.245 (0.400) |
|  |  |  |  |  |  |  |
| Standard CRP (mg/dL) | 10.906 (0.114) | 148.293 (0.227) | 11.317 (0.126) | 157.649 (0.251) | 7.982 (0.261) | 68.769 (0.517) |
|  |  |  |  |  |  |  |
| Period II |  |  |  |  |  |  |
|  |  |  |  |  |  |  |
| FIRI (μIU/mL) | 2.521 (0.079) | 9.297 (0.158) | 2.953 (0.088) | 13.495 (0.175) | 1.580 (0.180) | 2.829 (0.357) |
|  |  |  |  |  |  |  |
| UAE (mg/gCr) | 18.432 (0.079) | 387.471 (0.158) | 17.655 (0.088) | 362.303 (0.175) | 12.579 (0.180) | 165.274 (0.357) |
|  |  |  |  |  |  |  |
| Serum cystatin C (mg/L) | 0.981 (0.176) | 2.105 (0.351) | 0.441 (0.196) | 0.255 (0.390) | 1.262 (0.388) | 1.690 (0.759) |
|  |  |  |  |  |  |  |
| hs-CRP (ng/ml) | 6.840 (0.184) | 53.365 (0.365) | 4.179 (0.206) | 20.180 (0.408) | 3.570 (0.393) | 12.533 (0.768) |
|  |  |  |  |  |  |  |
| Standard CRP (mg/dL) | 8.459 (0.112) | 81.213 (0.224) | 8.790 (0.124) | 87.016 (0.247) | 6.613 (0.258) | 46.335 (0.511) |
|  |  |  |  |  |  |  |

Values are means (SE). BMI, body mass index; FPG, fasting plasma glucose; HbA1c, glycated hemoglobin A1c; TG, serum triglycerides; HDL-C, serum high-density-lipoprotein cholesterol; TC, serum total cholesterol; SBP, systolic blood pressure; DBP, diastolic blood pressure; AST, serum aspartate aminotransferase; ALT, serum alanine aminotransferase; GGTP, serum gamma-glutamyltransferase; ChE, serum cholinesterase; FLUS, fatty liver using ultrasonography scores; JPDRISC, Japanese Diabetes Risk Score; FIRI, serum fasting immunoreactive insulin; UAE, urinary albumin excretion; hs-CRP, serum high-sensitivity C-reactive protein; CRP, serum C-reactive protein.

**Supplemental Table S3.** Spearman's rank-order correlation between FIRI in Period II and variables in Period I.

| Parameter　(Reference Range and Unit) | All people | | People with FPG<100 mg/dl | | People with FPG≥100 mg/dl | |
| --- | --- | --- | --- | --- | --- | --- |
|  |  |  |  |  |  |  |
|  | rs | *p* | rs | *p* | rs | *p* |
|  |  |  |  |  |  |  |
|  |  |  |  |  |  |  |
| Period I |  |  |  |  |  |  |
|  |  |  |  |  |  |  |
| Age (years) | -0.013 | 0.680 | -0.066 | 0.066 | -0.071 | 0.337 |
|  |  |  |  |  |  |  |
| BMI (18.5-24.9kg/m^2^) | 0.501 | <0.001 | 0.469 | <0.001 | 0.449 | <0.001 |
|  |  |  |  |  |  |  |
| FPG (65-109mg/dL) | 0.305 | <0.001 | 0.237 | <0.001 | 0.036 | 0.627 |
|  |  |  |  |  |  |  |
| HbA1c (4.3-5.8%) | 0.170 | <0.01 | 0.084 | 0.029 | 0.127 | 0.095 |
|  |  |  |  |  |  |  |
| Waist Circumference (cm) | 0.525 | <0.001 | 0.505 | <0.001 | 0.480 | <0.001 |
|  |  |  |  |  |  |  |
| TG (30-149mg/dL) | 0.402 | <0.001 | 0.346 | <0.001 | 0.488 | <0.001 |
|  |  |  |  |  |  |  |
| HDL-C (40≤ mg/dL) | -0.350 | <0.001 | -0.316 | <0.001 | -0.382 | <0.001 |
|  |  |  |  |  |  |  |
| TC (120-219mg/dL) | 0.150 | <0.001 | 0.124 | 0.001 | 0.174 | 0.019 |
|  |  |  |  |  |  |  |
| SBP (90-129mmHg) | 0.280 | <0.001 | 0.255 | <0.001 | 0.212 | 0.004 |
|  |  |  |  |  |  |  |
| DBP (50-84mmHg) | 0.268 | <0.001 | 0.246 | <0.001 | 0.148 | 0.046 |
|  |  |  |  |  |  |  |
| AST (10-30U/L) | 0.268 | <0.001 | 0.234 | <0.001 | 0.304 | <0.001 |
|  |  |  |  |  |  |  |
| ALT (6-30U/L) | 0.400 | <0.001 | 0.363 | <0.001 | 0.427 | <0.001 |
|  |  |  |  |  |  |  |
| GGTP (15-50U/L) | 0.359 | <0.001 | 0.328 | <0.001 | 0.319 | <0.001 |
|  |  |  |  |  |  |  |
| ChE (200-450U/L) | 0.406 | <0.001 | 0.360 | <0.001 | 0.401 | <0.001 |
|  |  |  |  |  |  |  |
| FLUS (points) | 0.419 | <0.001 | 0.366 | <0.001 | 0.436 | <0.001 |
|  |  |  |  |  |  |  |
| Total JPDRISC (points) | 0.294 | <0.001 | 0.240 | <0.001 | 0.314 | <0.001 |
|  |  |  |  |  |  |  |
| Standard CRP (mg/dL) | 0.236 | <0.001 | 0.207 | <0.001 | 0.325 | 0.002 |
|  |  |  |  |  |  |  |

P <0.05 was considered significant. FIRI, fasting immunoreactive insulin; rs, Spearman's correlation coefficient; BMI, body mass index; FPG, fasting plasma glucose; HbA1c, glycated hemoglobin A1c; TG, serum triglycerides; HDL-C, serum high-density-lipoprotein cholesterol; TC, serum total cholesterol; SBP, systolic blood pressure; DBP, diastolic blood pressure; AST, serum aspartate aminotransferase; ALT, serum alanine aminotransferase; GGTP, serum gamma-glutamyltransferase; ChE, serum cholinesterase; FLUS, fatty liver using ultrasonography scores; JPDRISC, Japanese Diabetes Risk Score; CRP, serum C-reactive protein.

**Supplemental Table S4.** Spearman's rank-order correlation between UAE in Period II and variables in Period I.

| Parameter　(Reference Range and Unit) | All people | | People with FPG<100 | | People with FPG≥100 | |
| --- | --- | --- | --- | --- | --- | --- |
|  |  |  |  |  |  |  |
|  | rs | *p* | rs | *p* | rs | *p* |
|  |  |  |  |  |  |  |
|  |  |  |  |  |  |  |
| Period I |  |  |  |  |  |  |
|  |  |  |  |  |  |  |
| Age (years) | 0.215 | <0.001 | 0.156 | <0.001 | 0.252 | 0.001 |
|  |  |  |  |  |  |  |
| BMI (18.5-24.9kg/m^2^) | 0.106 | 0.001 | 0.024 | 0.513 | 0.244 | 0.001 |
|  |  |  |  |  |  |  |
| FPG (65-109mg/dL) | 0.157 | <0.001 | 0.040 | 0.267 | 0.171 | 0.021 |
|  |  |  |  |  |  |  |
| HbA1c (4.3-5.8%) | 0.163 | <0.001 | 0.088 | 0.021 | 0.215 | 0.004 |
|  |  |  |  |  |  |  |
| Waist Circumference (cm) | 0.135 | <0.001 | 0.049 | 0.170 | 0.288 | <0.001 |
|  |  |  |  |  |  |  |
| TG (30-149mg/dL) | 0.100 | 0.002 | 0.038 | 0.289 | 0.200 | 0.007 |
|  |  |  |  |  |  |  |
| HDL-C (40≤ mg/dL) | -0.060 | 0.062 | -0.022 | 0.534 | -0.099 | 0.184 |
|  |  |  |  |  |  |  |
| TC (120-219mg/dL) | 0.095 | 0.003 | 0.042 | 0.245 | 0.205 | 0.005 |
|  |  |  |  |  |  |  |
| SBP (90-129mmHg) | 0.225 | <0.001 | 0.155 | <0.001 | 0.343 | <0.001 |
|  |  |  |  |  |  |  |
| DBP (50-84mmHg) | 0.237 | <0.001 | 0.171 | <0.001 | 0.320 | <0.001 |
|  |  |  |  |  |  |  |
| AST (10-30U/L) | 0.092 | 0.004 | 0.060 | 0.095 | 0.139 | 0.061 |
|  |  |  |  |  |  |  |
| ALT (6-30U/L) | 0.083 | 0.010 | 0.026 | 0.463 | 0.158 | 0.033 |
|  |  |  |  |  |  |  |
| GGTP (15-50U/L) | 0.082 | 0.011 | 0.009 | 0.796 | 0.176 | 0.017 |
|  |  |  |  |  |  |  |
| ChE (200-450U/L) | 0.064 | 0.083 | 0.017 | 0.686 | 0.060 | 0.443 |
|  |  |  |  |  |  |  |
| FLUS (points) | 0.146 | <0.001 | 0.130 | 0.002 | 0.071 | 0.369 |
|  |  |  |  |  |  |  |
| Total JPDRISC (points) | 0.165 | <0.001 | 0.097 | 0.013 | 0.307 | <0.001 |
|  |  |  |  |  |  |  |
| Standard CRP (mg/dL) | 0.002 | 0.961 | -0.017 | 0.746 | -0.001 | 0.992 |
|  |  |  |  |  |  |  |

P <0.05 was considered significant. UAE, urinary albumin excretion; rs, Spearman's correlation coefficient; BMI, body mass index; FPG, fasting plasma glucose; HbA1c, glycated hemoglobin A1c; TG, triglyceride; HDL-C, high-density-lipoprotein cholesterol; TC, total cholesterol; SBP, systolic blood pressure; DBP, diastolic blood pressure; AST, aspartate aminotransferase; ALT, alanine aminotransferase; GGTP, gamma-glutamyltransferase; ChE, cholinesterase; FLUS, fatty liver using ultrasonography scores; JPDRISC, Japanese Diabetes Risk Score; CRP, C-reactive protein.

**Supplemental Table S5.** Spearman's rank-order correlation between hs-CRP in Period II and variables in Period I.

| Parameter　(Reference Range and Unit) | All people | |
| --- | --- | --- |
|  |  |  |
|  | rs | *p* |
|  |  |  |
|  |  |  |
| Period I |  |  |
|  |  |  |
| Age (years) | 0.001 | 0.987 |
|  |  |  |
| BMI (18.5-24.9kg/m^2^) | 0.449 | <0.001 |
|  |  |  |
| FPG (65-109mg/dL) | 0.146 | 0.054 |
|  |  |  |
| HbA1c (4.3-5.8%) | 0.194 | 0.016 |
|  |  |  |
| Waist Circumference (cm) | 0.505 | <0.001 |
|  |  |  |
| TG (30-149mg/dL) | 0.397 | <0.001 |
|  |  |  |
| HDL-C (40≤ mg/dL) | -0.355 | <0.001 |
|  |  |  |
| TC (120-219mg/dL) | 0.106 | 0.164 |
|  |  |  |
| SBP (90-129mmHg) | 0.201 | 0.008 |
|  |  |  |
| DBP (50-84mmHg) | 0.186 | 0.014 |
|  |  |  |
| AST (10-30U/L) | 0.222 | 0.003 |
|  |  |  |
| ALT (6-30U/L) | 0.342 | <0.001 |
|  |  |  |
| GGTP (15-50U/L) | 0.377 | <0.001 |
|  |  |  |
| ChE (200-450U/L) | 0.335 | <0.001 |
|  |  |  |
| FLUS (points) | 0.415 | <0.001 |
|  |  |  |
| Total JPDRISC (points) | 0.228 | 0.003 |
|  |  |  |
| Standard CRP (mg/dL) | 0.468 | <0.001 |
|  |  |  |

P <0.05 was considered significant. hs-CRP, serum high-sensitivity C-reactive protein; rs, Spearman's correlation coefficient; BMI, body mass index; FPG, fasting plasma glucose; HbA1c, glycated hemoglobin A1c; TG, triglyceride; HDL-C, high-density-lipoprotein cholesterol; TC, total cholesterol; SBP, systolic blood pressure; DBP, diastolic blood pressure; AST, aspartate aminotransferase; ALT, alanine aminotransferase; GGTP, gamma-glutamyltransferase; ChE, cholinesterase; FLUS, fatty liver using ultrasonography scores; JPDRISC, Japanese Diabetes Risk Score; CRP, C-reactive protein.

**Supplemental Table S6.** Linear regression coefficients (*β*) for the association between parameters measured in Period I and high-sensitivity C-reactive protein in Period II.

|  | **All people** | | |
| --- | --- | --- | --- |
| Complete pairs of dataset | N=120 | | |
| Parameters | *β* | Standardized *β* | *P* |
|  | (95% CIs of *β*) |  |  |
| ALT (U/L) | -18.023 | -0.105 | 0.250 |
|  | (-48.915 – 12.868) | |  |
| FLUS (points) | 1125.650 | 0.258 | 0.008 |
|  | (304.330 – 1946.970) |  |  |
| JPDRISC (points) | -39.114 | -0.042 | 0.628 |
|  | (-198.555 – 120.326) | |  |
| ChE (U/L) | 0.238 | 0.005 | 0.952 |
|  | (-7.599 – 8.075) |  |  |
| GGTP (U/L) | 4.455 | 0.070 | 0.432 |
|  | (-6.733 – 15.643) | |  |
| TG (mg/dL) | 15.217 | 0.585 | <0.001 |
|  | (10.677 – 19.756) |  |  |
| TC (mg/dL) | -14.426 | -0.160 | 0.057 |
|  | (-29.268 – 0.416) |  |  |
| DBP (mmHg) | 4.705 | 0.013 | 0.877 |
|  | (-55.259 – 64.669) |  |  |
| FPG (mg/dL) | -3.846 | -0.025 | 0.766 |
|  | (-29.373 – 21.680) |  |  |
| Constant | 2074.642 | NA | 0.441 |
|  | (-3243.814 – 7393.098) | |  |

*β*, regression coefficient; CIs, confidence intervals; ALT, serum alanine aminotransferase; FLUS, fatty liver using ultrasonography scores; JPDRISC, Japanese Diabetes Risk Score; ChE, serum cholinesterase; GGTP, serum gamma-glutamyltransferase; TG, serum triglycerides; TC, serum total cholesterol; DBP, diastolic blood pressure; FPG, fasting plasma glucose; NA, not applicable. Factors of age and body mass index were included into JPDRISC.

**Supplemental Table S7.** Linear regression coefficients (*β*) for the association between parameters measured in Period II and high-sensitivity C-reactive protein in Period II.

|  | **All people** | | |
| --- | --- | --- | --- |
| Complete pairs of dataset | N=156 | | |
| Parameters | *β* | Standardized *β* | *P* |
|  | (95% CIs of *β*) |  |  |
| ALT (U/L) | -26.777 | -0.173 | 0.101 |
|  | (-58.799 – 5.245) | |  |
| FLUS (points) | 529.017 | 0.146 | 0.105 |
|  | (-112.481 – 1170.516) | |  |
| JPDRISC (points) | 0.21 | 0.002 | 0.977 |
|  | (-14.165 – 14.588) | |  |
| ChE (U/L) | -0.089 | -0.002 | 0.979 |
|  | (-6.678 – 6.499) |  |  |
| GGTP (U/L) | 3.743 | 0.056 | 0.517 |
|  | (-7.633 – 15.120) | |  |
| TG (mg/dL) | 14.622 | 0.465 | <0.001 |
|  | (8.816 – 20.427) |  |  |
| TC (mg/dL) | -13.302 | -0.163 | 0.055 |
|  | (-26.879 – 0.276) |  |  |
| DBP (mmHg) | 24.520 | 0.075 | 0.360 |
|  | (-28.224 – 77.264) |  |  |
| FPG (mg/dL) | 10.729 | 0.065 | 0.439 |
|  | (-16.595 – 38.053) |  |  |
| Constant | -398.036 | NA | 0.869 |
|  | (-5151.124 – 4355.052) | |  |

*β*, regression coefficient; CIs, confidence intervals; ALT, serum alanine aminotransferase; FLUS, fatty liver using ultrasonography scores; JPDRISC, Japanese Diabetes Risk Score; ChE, serum cholinesterase; GGTP, serum gamma-glutamyltransferase; TG, serum triglycerides; TC, serum total cholesterol; DBP, diastolic blood pressure; FPG, fasting plasma glucose; NA, not applicable. Factors of age and body mass index were included into JPDRISC in this analysis.
